# Neurasthenic and Hysterical Cases in General Military Hospitals

**Published:** 1919

**Authors:** 


### NEUROLOGY AND PSYCHIATRY

---

***Neurasthenic and Hysterical Cases in General Military Hospitals.*** By R. H. Trotter, Major R. A. M. C. *The Lancet*, November 23, 1918.

The author, convinced of the necessity for early diagnosis and treatment, urges prompt measures, as habit speedily becomes fixed in this class of patients and is difficult to break. The present tendency is to diagnose the case, refer it to some neurological center, and take no further interest in it. There is also a disposition to exalt surgery at the expense of medicine, and to lose sight of the patient in the perfection of technic.

Though for continued treatment and complete cure of nearly all psychical neuroses, the mental atmosphere and open-air treatment are essential, emergency treatment for these patients should be available in every general hospital. These are : accurate diagnosis, confident prognosis, understanding sympathy and inexhaustible patience. Given these, many cases may be cured at once, and others placed on the high road to recovery.

*Treatment* : The patient is partially isolated, but irksome restriction is avoided. After diagnosis his condition is explained as far as he can understand it, and he is confidently assured of recovery. If motor psychosis takes a spastic form, passive motion is practiced until the muscles relax; if the " palsy " is flaccid, manipulate until a flicker of muscular contraction takes place, constantly assuring the patient that he will get all right. At the least sign of muscular contraction he is urged to resist the passive movements, and often hours elapse before muscular resistance is sufficient to retain the "palsied" limb momentarily rigid; a sudden interruption then often surprises the patient into maintaining the limb in the posture in which it has been abandoned. From this point it is easier to convince the patient that the limb is not paralyzed. The principal danger is that he will acquire a fantastic gait — this can be avoided by giving him a little supervision, exercise and employment.

Psychical neuroses can frequently be overcome by a psychoanalytical talk, which may lead the patient to a normal outlook on life. Unceasing vigilance is called for in the surgical ward to prevent development of neuropathic conditions.

Cases cited cover pleurisy and acute rheumatism, with endocarditis, myalgia, intestinal obstruction, paralysis of both lower limbs and left arm, fractured pelvis, and mutism, with recovery in every instance under prompt, understanding and sympathetic treatment.

